# Faith-based organizations: humanitarian mission or religious missionary

**DOI:** 10.1186/s41018-020-00080-6

**Published:** 2020-10-09

**Authors:** Riham Ahmed Khafagy

**Affiliations:** Department of International Studies, College of Humanities and Social Sciences, Zayed University, Academic City, P.O. BOX: 19282, Dubai, United Arab Emirates

**Keywords:** Humanitarian relief, Direct Aid Society, Islamic Relief Worldwide, Islamic organizations, Faith-based organizations, Religious organizations

## Abstract

This paper analyzes Islamic FBOs’ humanitarian approaches, programs, and challenges. Politicalized religious interpretations are also on board to investigate their missionary aspects. I design my argument based on Michael Barnett and Janice Grass Stein’s assumption on the impact of social constructions on establishing sacred and secular concepts and spaces. Thus, I study the UK-based Islamic Relief Worldwide (IRW) and the Kuwaiti Direct Aid Society (DAS) to examine the influence of their social settings on their humanitarian experiences. My question is “do different social settings shape various humanitarian approach, although of sharing the same religious mission?” I argue that Islamic rules encourage Muslims to be religiously committed to paying charity and showing human and religious solidarity. In this regard, flexible Islamic *Fiqh* (jurisprudence) allows Muslima to set various socially constructed implementations of these religious commitments. Humanitarian relief is not an exception in this regard. Therefore, Islamic FBOs lay down on a continuum based on their socially constructed models which reflect different interpretations of religious texts and their applications to understanding societal issues as well as various employed strategies of these civil society actors.

## Introduction

Since the mid-1980s, faith-based organizations (FBOs) have increasingly involved in humanitarian relief. They enjoy committed volunteers, plenty of donations, and access to underdeveloped communities. Alongside the presence of other FBOs, Islamic and Christian organizations became influential actors in the international arena. They vary in their institutional structure, financial resources, or interests; some of them collect donations, while others are funded by religious entities. They either run by religiously committed volunteers or hire professional staff. However, they share a religious mission with caring traditions and long-lasting societal impacts. Frequently linked to the increasing politicization of religion, FBOs challenge blurry borders between “humanitarian mission” and “religious missionary” as well as revise dominant secular settings of humanitarian relief.

In this paper, I analyze Islamic FBOs’ humanitarian approaches, programs, and challenges. Politicalized religious interpretations are also on board to investigate their missionary aspects. Theoretically, Michael Barnett and Janice Grass Stein argue that secularization and sanctification are multilayered, multidimensional, and nonlinear. The historically constructed process comes first to establish sacred and secular concepts and spaces. Then, setting, stabilizing, or modifying them might be a strategy that is employed by different actors to further their agendas (Barnett and Stein 2012: 11). It is not only about religious rules but also social contexts matter. Based on this argument, I study two Islamic FBOs’ humanitarian relief to trace the impacts of different social constructions on shaping their approaches and programs, while sharing the same religious mission.

The two studied cases are the UK-based Islamic Relief Worldwide (IRW) and the Kuwaiti Direct Aid Society (DAS). Although based in different countries, both are active Islamic humanitarian organizations. I examine the influence of their social settings on their humanitarian approaches and experiences. My question is “do different social settings shape various humanitarian approaches, although of sharing the same religious mission?” I argue that Islamic rules encourage Muslims to be religiously committed to paying charity and showing human and religious solidarity. In this regard, Islamic *Fiqh* (jurisprudence) flexibility allows Muslims to set various socially constructed implementations of these religious commitments. Humanitarian relief is not an exception in this regard. Islamic FBOs lay down on a continuum; various models reflect different interpretations of religious texts and their applications to understanding societal issues as well as several employed strategies of these civil society actors. Therefore, I divided my paper into two sections. The first one explores the theoretical, socioeconomic, and political aspects of FBO, with a special emphasis on the Islamic ones. The second section elaborates on the two Islamic FBOs case studies; empirically compare their backgrounds, developmental and humanitarian approaches, and executive programs to examine differences and similarities of their visions and actions. Finally, the conclusion predicts the potential flourishing or falling of Islamic FBOs’ roles.

## First section: Islamic FBOs: theoretical, socioeconomic, and political aspects

During the last two decades, social science scholars and civil society activists have expressed an increasing interest in investigating FBOs’ roles (Kemper 2006: 141-153). Numerous articles and research papers sought to define the concept of FBOs; question their goals; and evaluate their effectiveness, subjectivity, and religiosity. Scholars debate several formulas for potential inclusion or exclusion of these organizations in contemporary humanitarian relief and developmental activities (Bielefeld and Cleveland 2013: 445-446). This section aims to contribute to this debate throughout discussing the remarkable theoretical, socioeconomic, and political aspects of FBOs, with special emphasis on the Islamic model.

### Theoretical aspects—religion in public humanitarian sphere

After decades of disconnecting religious faith from the larger public sphere, social processes have gradually opened a space for the resurgence of religious life in contemporary society. Legally connected, but culturally fragmented societies have tried to develop shared values and norms and consequently establish communal institutions. Religious faiths seem like one of the rare traditions that could shape a similar collective sense and suggest a base for its relevant actions. Thus, the Western historically established privatization of religious practices is increasingly challenged by a robust tendency toward a public reconsideration of religious ties. Simultaneously, neoliberal policies overload individuals with social and economic responsibilities, while significantly eliminate the state’s public funding. Within this context, religious structures eagerly run to fill this gap, and rapidly expand their influence in social, political, and humanitarian fields.

Charles Taylor classifies three major processes that are necessary for achieving secularization in any society; declining belief in God, decreasing the importance of religion and religious organizations, and developing changed conditions of belief (Harding 2013: 342). The first process reflects a personal choice, in which individuals enjoy the freedom of privatizing their religious faith or simply giving it up. The other two processes are socially constructed, as being inherited from historical traditions and integrated into daily practices. So, the inclusion of new social groups with diverse values and desires might re-question settled legacies and arrangements. The snowballing multicultural immigration flux has introduced the European societies to various heritages of the conditions of belief. Multiculturalism has paved a way to a timid revival of the belief in God, so religious organizations have enhanced their social presence and influence. This slowly develops several socially constructed versions of secularization and scarification in Western societies (Barnett and Stein 2012: 10).

Barnett and Grass Stein define secularization of humanitarianism as “the process by which elements of every day and the profane insinuate themselves and become integrated into humanitarianism, thus challenging its scared standings” (Barnett and Stein 2012: 8). This process of secularization requires a growing role of the state and the market, implies a centrality of the fund, and expects an effective and efficient performance from humanitarian relief organizations. Recently, many of these requirements have gradually declined in contemporary European societies to be replaced by various levels of sanctification. Per Barnett and Grass Stein, sanctification is a free space of politics, inspired primarily by humanitarian values and motivated purely by innocent and altruistic incentives, which could apply to religious organizations. Both secularization and sanctification evolve in historically dynamic ways to shape trends, practices, and tensions of humanitarianism (Barnett and Stein 2012: 8).

Secular humanitarian organizations are by no means less ethical than their religious counterparts. However, the former seems to be less missionary; more professional, and considerably supported by their states or official regional or international institutions. The latter is generally missionary based; its professionality is less emphasized and is mostly energized by communal voluntary arrangements. Religious humanitarianism refers to actions that go beyond traditional social-relational activities. It clearly states religious incentives, implies faith-friendly mechanisms, and partially assesses its works considering religious obligations and rewards.

Investigating the Swedish community, Tobias Harding observes a growing interest influence of various religious faiths, including the newly introduced ones by immigrants. They used to be recognized by the state as domestic multiculturalism, but the Swedish neo-corporative system allows ethnic associations and FBOs an expanding role in delivering welfare services. Per Harding, this role is a turning point of contemporary domestic societal mechanisms (Harding 2013: 344). More broadly, Alastair Ager describes the growing involvement of FBOs and local faith communities in humanitarian relief as a “post-secular age.” Referring to their religious faiths, these organizations challenge the dominant humanitarian values and norms. Settled humanitarian values have been articulated based on secular consistent concepts. Thus, FBOs’ struggle to strictly follow these values and norms might neither fulfill their volunteers’ requests nor satisfy recipient communities’ expectations (Ager 2014: 17-18)^1^.

From another perspective, Khaled Mansour and Heba Raouf Ezzat criticize the over-simplistic differentiation between FBOs and secular societal organizations. Per them, any distinction between both humanitarian institutional forms merely reflects certain social and historical experiences (Mansour and Ezzat, 2009: 123, 127). Such a debate used to be irrelevant to the traditions of most of the non-Western societies. Due to the rapid modernization and intensive globalization of these societies, they have recently recognized this debate, but still with limited domestic consequences. Mansour and Ezzat argue that many active members in Islamic FBOs denounce secularization of humanitarian relief as targeting to deprive the religious identity of their activities (Mansour and Ezzat 2009: 123, 127). Yet, this debate sounds very relevant to Western-based Islamic FBOs’ roles, especially those are done in Western and non-Western societies, and challenged by different setting alongside various expectations.

In the Islamic model, religious jurisprudence allows a flexible understanding of the *Holy Qur’an* and the prophet’s sayings and deeds, as main sources of Islamic rules, to accommodate changeable realities. The *Ultimate* goals of the Islamic *Shariah* (rules) are to preserve the life, the wealth, the human reason, the human species, and the honor or dignity of all humanity. Some interpretations understand the last goal as the preservation of the religion itself. These goals set a comprehensive and universal humanitarian mission for Muslims. Only a few compulsory rules are mentioned in the *Holy Qur’an*. Otherwise, Islamic jurisprudence sets the concepts of *Maslaha* (interest) and *Mafsadah* (corruption) to evaluate each deed and flexibly balance its religious rewards or punishment considering contemporary contexts. The level of the sanctification of each action varies and actual priorities create their relevant sacred duties. Lacking any hierarchical religious institutions in the Islamic traditions, especially the *Sunni*, encourages constructive diversity of interpretations of religious texts and the implementations of relevant commitments.

Conceptually, the widely used definition of FBOs are “those humanitarian relief and development organizations formed by or with a direct or indirect relationship to a specific faith community.” Islamic FBOs, especially *Sunni* ones, could be slightly different. They are usually launched by individuals’ independent initiatives, and in few cases, governmental patronage or support might come second. While the Islamic *Shi’ai* hierarchical structure facilitates the establishment of many *Shi’ai* FBOs; each is closely connected to a certain *Shi’ai Hawza* (seminary) and enjoys its financial patronage. Few *Shi’ai* organizations independently established themselves, like the Aga Khan Foundation. Although its founder is the head of the *Ismaili Shi’ai*, the Foundation introduces itself as a secular entity. *Sunni* and *Shi’ai* FBOs are generally committed to implementing Islamic rules. I believe further research is needed to compare various cases of *Sunni* and *Shi’ai* FBOs, and investigate their common or dissimilar characteristics considering their similar religious faith, but belonging to different sects. However, I focus in this paper on two Islamic FBOs that share the same *Sunni* background to avoid any variances in their referred theology during the process of testing my hypothesis.

Bruno De Cordier suggests a definition of Islamic FBOs, as “non-governmental organizations (NGOs) that were founded on the initiative of Muslims, that mobilize most of their support among Muslims, and whose action is, to varying degrees and in various forms, inspired and legitimated by the Islamic religion or at least certain tenets thereof” (De Cordier 2009: 609). His definition noticeably explains these organizations’ activities, while it misses their religious agenda. Less attention is paid to clarify their religious mission and goals. Consistently, it calls them “Muslims Faith-Based organizations” to emphasize on their active participants instead of describing them as “Islamic organizations” and connecting them directly to the religious faith (De Cordier 2009: 609). I think that Islamic FBOs are better defined as “Faith inspired organizations which are instituted with a mission statement informed by generalized spiritual principals, perhaps of one of the major world religions, but which are founded and run independently of any specifically identified faith community” 2. For Islamic cases, differences between faith-inspired organizations and FBOs are sometimes blurred.

### Socioeconomic aspects—always here and there

Historically, religious communities used to fulfill societal needs throughout charitable activities and humanitarian relief. Only since the nineteenth century, major secular humanitarian organizations have been established, with a substantial expansion in the twentieth century. The Red Cross and anti-slavery organizations are only a couple of centuries old. UNHCR was a sign of an intergovernmental organization in the 1960s (Ferris 2005: 313-316). Simultaneous with the European secularization, FBOs have been marginalized in the international humanitarian relief, while they have maintained their influential developmental roles in non-Western communities. Since the early 1980s, these communities have witnessed a mushrooming expansion of their local non-governmental organizations (NGOs), including FBOs. Due to sharing similar concerns and cultural understandings, local FBOs successfully involved in several societal projects and enjoyed convenient access to their local communities (Ferris 2005: 316). Various religious faiths established FBOs, like Islamic, Hundi, Christian, and Jewish cases (Mansour and Ezzat 2009: 118).

Academic comparison between NGOs and FBOs concluded that both models provide social services at almost a similar quality. FBOs are, theoretically, classified within the gray area between NGOs and religious organizations. Paving a new path, they integrate humanitarian incentives as well as religious commitments; they could conduct NGOs’ civic roles with a religious added value^3^. This is hardly related to the level of the ethicality of each model, but it is mainly linked to their different missions. Further research is recommended to deeply compare between both missions and trace their humanitarian and developmental approaches and programs. FBOs can recruit religiously-dedicated volunteers and secure financial resources from religious charities. While NGOs have increasingly sought domestic or international governmental funds, which questioning their non-governmentality (Ebaugh et al. 2003: 411-426). Additionally, FBOs enjoy coherent management structures and suffer less organizational weakness comparing to their NGO counterparts (Clerkin and Grønbjerg 2007: 115-126).

Religious organizations, especially in the non-Western communities, are deeply rooted and well informed about their societal contexts. So, they can diagnose causal factors behind most of their fundamental challenges. Rising poverty, high unemployment rates, and wide social exploitation have motivated the establishment of various FBOs in many underdeveloped countries. They show deep social commitment, cultural sensitivity, and convenient accessibility to different social classes. Based on their religious backgrounds, they also form coherent vision about required changes and expected outcomes. FBO positively run their activities alongside a constructive governmental role and in a welcoming society. Multilayer social evils, disputed actors, fragmented political approaches, and social resistance hinder FBOs’ effectiveness in achieving their targeted social change. Rick James acknowledges FBOs as “providing efficient development services; reach the poorest at the grassroots; having a long term sustainable presence, being legitimate and values by the poorest, providing an alternative to secular theory for development, eliciting motivated and voluntary service, encouraging civil society advocate” (James, 2011: 111).

Empirical studies stress the significance of psychological support alongside material relief for people in war-torn or post-conflict societies, or even after a natural catastrophe (Barnett and Stein 2012: 22; Act Alliance 2015: 1; (James, 2011: 113-114)). Inspired by their religious missions, FBOs offer spiritual assistance for their targeted beneficiaries, alongside the materialistic support. They show respect for hosting communities’ faiths and seek local consensus on controversial social issues, such as women’s rights or HIV/AIDS. Neglecting such actions could jeopardize the efficiency and effectiveness of any relief tasks (Kirmani et al. 2008: 8). For example, Muslim and Christian religious leaders played influential roles in the response to epidemic Ebola in West African countries. They adopted a holistic approach to replace the message of fear with a message of hope, shape new attitudes toward affected people, and transform physical and spiritual practices of local community members (Featherstone 2015: 9-10). Similar roles are usually expected and welcomed regarding HIV/AIDS, most recently the pandemic COVID-19. FBOs frequently invest religious local leaders’ support to facilitate achieving their goals.

In the Islamic case, religious rules explicitly recommend conducting as well as funding societal services and developmental goals. Financially capable Muslims must pay *Zakat* (regulated obligatory charity) and are encouraged to donate *Sadaqat* (unlimited voluntary charity)^4^. Both support activities aim to satisfying Allah as well as showing human solidarity. Although priority is given for serving Muslims, non-Muslims are also covered. *Waqf* (endowment) is an institutionalized form of *Sadaqat Garyia* (running and sustainable voluntary charity). Widely established Islamic endowments effectively contributed to delivering social services in their local communities, such as education, social care, health services, and humanitarian relief. Historically, these financially independent endowments secured most of the required infrastructure of their societies and offered sustainable developmental services. Contemporary Islamic FBOs are guided by Islamic teachings as well as inspired by *Waqf’s* historical experiences (Mansour and Ezzat 2009: 120). Both are religiously inspired and targeted humanitarian and developmental goals. Like *Waqf*, Islamic FBOs enjoy considerable financial independence and devoted volunteers. Governmentally funded FBOs, either supported by local governments or intergovernmental organizations, frequently suffer political and logistical challenges^5^. Lacking any Islamic hierarchal religious institutions also allows FBOs significant autonomy in accommodating religious commitments to fit recipient societies^6^.

### Political aspects—neoliberalism and terrorism

The increasing interest in FBOs’ humanitarian and developmental roles has been associated with the broad execution of neoliberal policies as well as the increasing terrorist attacks.

#### Neoliberalism

Gerard Clarks traces the evolving importance of FBOs back to the early 1990s following the publication of the World Bank study “Voices of Poor,” which acknowledged their developmental activities. More appreciation was expressed later by the World Bank administration and the UK Department of International Development to encourage enhancing these roles (Clarke 2007: 81-83). British motivating policies were followed by the Clinton’s charitable choice provision in 1996 and the Bush’s faith-based initiative in 2001. Both allow FBOs to be eligible for the federal fund, without requiring suppressing or concealing their religious identity or practices (Carlson-Thies 2009: 936). Under George W. Bush’s administration, American FBO received federal grants totaling more than ten billion dollars. Obama’s version of the faith-based initiative maintained most of Bush’s rules. In early May 2018, President Donald Trump announced an executive order to expand the allocation of the government grants and the arranged partnerships with FBOs through a newly established faith-based office (Bailey and Boorstein 2018).

These procedures frequently question the secularization process of Western societies and the level of religiosity of delivering their social services (Anti-Defamation League 2012: 1-5). Neoliberalism intensively encourages the market based providing of social services. In this regard, reliable substitutes of the state’s role were needed to avoid the serious consequences of these policies. FBOs practically enjoy accessibility in inner communities far more than the state or the market. Thus, George W. Bush and Tony Blair described sometimes as religiously inspired politicians, facilitated the replacement of these organizations’ societal interventions to a shrinking state’s role (Zehavi 2008, 347). Jeffrey C. Issac criticizes these initiatives and stresses that a promising contribution by civil society to public policy in the post-welfare state should address structural social and economic challenges, raise fundamental issues, question statist policies, and oppose unregulated capitalist markets (Issac 2003: 9). Supposedly, FBOs should seek profound social change, instead of contributing to the current system’s survival process.

So far, many FBOs have been integrated into the current international neoliberal system. However, with limited potential for changing the economic policies, they enhance the sanctification process of their social context. Unfortunately, religious hands have increasingly contributed to fixing neoliberal deficiencies and mitigating their societal consequences. Relevantly, Bruno De Cordier examines the “Mountain Societies Development Support Program (MDSP)”, which was initiated by the Aga Khan Foundation since 1993, and has operated humanitarian relief and developmental activities in an *Ismaili Shi’ites*-majority Tajiki providence. The program changed traditional economic structures in a way that De Cordier argues to benefit Aga Khan’s corporate. Bitterly, he criticizes such social and humanitarian programs that closely connected to religious leaders, while facilitating corporate’s control (De Cordier 2008: 170-172). Referring to all humanitarian organizations, Patrick Gibbons considers that “those organizations that continue to strive to priorities the needs of the victims of disasters and live by the traditional humanitarian principles are in the minority in this category” (Gibbons 2005: 12-13).

#### Terrorism

Simultaneous with the flourishing of the FBOs in the 1990s, Samuel Huntington predicted an outbreak of a clash among religiously classified civilizations, as religious legacies have a distinctive and lasting imprint in universal values. Thus, FBOs’ activities might be politically propagated, and economically interrelated with governments, multinational corporations, and international institutions’ interests (Mansour and Ezzat 2009: 123). Close to Barnett and Grass Stein’s argument, Jonathan Benthall and Jerome Bellion-Jourdan stress that religious legacies “are always selectively rebuilt to fulfill contemporary objectives” (Benthall and Bellion-Jourdan 2009: 154). Per them, this could apply to Christian as well as Islamic organizations. For Christian cases, they refer to the close relationship between the NATO forces and FBOs in Kosovo in 1999. Later, Christian evangelicals FBOs, especially Americans, were assigned to carry out emergency operations after the US-led military invasion in Afghanistan and Iraq in 2001 and 2003 respectively (Mansour and Ezzat 2009: 130-131).

Following the Islamic FBOs’ influential roles in the Afghan war (1978-1992) and Bosnia war (1992-1995), unproved accusations of their connections to terrorism have been firstly raised by Middle Eastern governments, especially Egypt and Saudi Arabia (Mansour and Ezzat 2009: 123-129). These governments aimed to weaken domestic Islamist political opposition by internationally restricting their humanitarian arms. After 9/11, Western governments widely accused most of the Islamic international FBOs of facilitating funding militant terrorism. Consequently, these governments adopted two strategies: chasing and coopting. Firstly, some of the accused organizations were shut down. Survival ones suffered tight restrictions on their money transfers and bank accounts and were enforced to conduct security clearance for their employees and submit detailed reports on their ongoing activities. The local governments had to impose these restrictions, regardless of their trust in targeted FBOs (Mansour and Ezzat 2009: 125-131).

Secondly, the cooption strategy was developed a few years later, encouraged the World Bank to revise its policies and seek active partnerships with FBO. Selected partner organizations included mainly Christian organizations and a few European-based Islamic ones. British and Dutch governments adopted a domestic similar strategy (Petersen: 2012: 5). Potential partnerships highly recommended that FBOs should achieve a significant alignment with the universal humanitarian values and professional standards. Thus, Islamic FBOs that sought to be involved in these partnerships had to adjust their speeches, goals, and programs to follow these recommendations. An accepted formula that integrated Islamic faith, international recommendations, and domestic settings was a challenge for each FBO.

## Second section: Islamic FBOs—different contexts, altered paths

I investigate backgrounds, humanitarian approaches, and programs of the two Islamic FBOs case studies; the Kuwaiti Direct Aid Society (DAS) and the UK-based Islamic Relief Worldwide (IRW). Based on Barnett and Stein’s argument on secularization and scarification, I compare these organizations’ interpretations of religious texts and implementations of their Islamic mission considering their different social contexts. By this, I seek to answer the question: “do different social settings shape various humanitarian approaches, although of sharing the same religious mission?” I argue that their different social contexts produce altered paths of their humanitarian relief efforts as well as their understandings of their Islamic “mission.” I selected these cases according to their programs’ geographical coverage and the level of governmental patronage.

For the first criterion, the DAS is active beyond its Kuwaiti local community and dominantly operates its programs in African countries. The IRW runs domestic and international humanitarian as well as developmental programs; contrarily to many Western-based Islamic FBOs which exclusively operate local community programs^7^. On the other side, neither the DAS nor the IRW is financially supported by any government. I excluded governmentally subsidized Islamic FBOs, such as the Saudi International Islamic Relief Organization (IIROSA), although it used to be one of the largest Islamic FBOs worldwide^8^. Such organizations suffer strict official censorship and are frequently accused of propagating states’ ideologies, such as Wahhabism/Salafism (Clarke 2007: 83-84). Selected cases secure their financial resources from charitable donations, mostly are religiously based, such as *Zakat* and *Sadaqat*. Both established their *Waqf* Islamic endowments to create a sustainable financial source for funding their operated programs. The IRW sometimes receives the British governmental grants that are allocated to support the civil society sector. Still, both should follow domestic regulations and auditing standards; and carefully consider their countries’ political and social sensitivities. None of them directly promotes their home country’s policies. However, throughout legalizing these organizations, the British and Kuwaiti governments could propagate their domestic policies and avoid critics for their international interventions. Profound opposition against governmental choices might hardly be developed within this context.

### Different contexts

The Kuwaiti Direct Aid Society (DAS) and the UK-based Islamic Relief Worldwide (IRW) share some significant characteristics of their backgrounds. Both were established in the mid-1980s as a response to a severe famine in Africa, and their founders were medical doctors, the Kuwaiti Abdul Rahman Al Sumit and the Egyptian-originated Hany El-Banna respectively. The DAS was primarily established in 1981, under the titled “Malawi Muslims Committee,” shortly later, it expanded its activities beyond Malawi. In 1984, the organization changed its name into the “Africa Muslim Committee.” Not until 1999, it got its current name as the “Direct Aid Society” (Direct Aid Society, 2019). Since its establishment, the organization’s humanitarian and developmental programs have benefited Muslims as well as non-Muslims in hosted communities. In parallel, the IRW was established in Birmingham, UK, in 1984, and launched various humanitarian programs in Africa, Iran, Afghanistan, and Iraq, alongside some domestic activities. Throughout the 1990s, it intensively contributed to humanitarian relief efforts in the Bosnian war. It was internationally acknowledged as a “moderate” and well-trusted Islamic FBO. Since 1994, it has been qualified to receive British governmental competitive grants and join numerous domestic and international partnerships, with governmental and non-governmental organizations. Additional to its various international branches, the IRW established few affiliated organizations under its umbrella but they are legally independent entities.

Charles Taylor distinguishes between different conditions of belief. A set of conditions is connected to a society in which the belief of God is a dominant approach. Another set of conditions is related to other societies in which irrelevant reasoning to the belief of God is expected in most contexts (Harding 2013: 342). I apply Taylor’s sets to the social contexts of the studied organizations. The Kuwaiti DSA is based on a conservative Muslim community, in which Islam is the dominant religion, and religious rituals and rules are habitually integrated into the public and private daily life. Proselytization to Islam is a favorable task and considered a religious commitment. A wide understanding of the *Ultimate* goals of Islamic *Shari’ah* refers to preserving human dignity by materialistic and spiritual methods. The secular universal language of humanity is not common because people believe that Islam is already a *universal* religion. Public interpretations of Islamic goals favor showing solidarity with other Muslims as a priority.

Kuwaiti regime partially gains its political legitimacy from being religiously conservative. The tribal local society has accumulated hung financial individual as well as governmental surpluses in a welfare state that subsidized social services. Thus, Kuwaiti Islamic FBOs target international communities in their humanitarian and developmental missions. Still, they domestically are welcomed and receive voluntary donations from the Royal Family and wealthy commercial elites. Volunteers are religiously-committed and motivated to support underprivileged communities suffering from conflicts or epidemic diseases. The DAS’s founder, Abdul Rahman Al-Sumit (1947-2013), spent most of his medical career (1983-2008) in Africa and stressed his religious and humanitarian “missions” there. However, since 2001, the Kuwaiti government has strictly imposed internationally required legal and financial revision and restrictions on local FBOs, including the DSA.

Within a different context, the IRW was established in Birmingham by a few Muslim immigrant doctors. An increasing significance of Muslim minorities has raised several inquiries about the challenging conditions of belief in God in Western secular societies, comparing to the case in Muslims-majority societies. The Islamic *Fiqh* (jurisprudence) of minorities has been developed to help Muslim immigrants understanding the *Ultimate* goals of Islamic *Shari’ah* considering their social contexts. Per Mansour and Ezzat expected interpretation of religious texts and their understanding of social issues should be “free from narrow, local considerations and the impact of community norms in respective countries on the limits of thinking about Islamic issues” (Mansour and Ezzat 2009: 142). Thus, this jurisprudence discusses secularized cultures and examines the privatization of religious faiths. It debates the Muslims’ inclusion in Western conditions of belief and their attempts to reach more sanctification of the public sphere. Four hundred constitutes and two hundred board members of American Muslim organizations were surveyed. They acknowledged the substantial impacts of their religious beliefs and humanitarian values on their decisions, while admitted that humanitarian “secular” reports are less influential in this regard. Other studies concluded with similar results for various immigrant groups; as they sought to maintain their cultural and religious identities more than being integrated into the American culture (Bielefeld and Cleveland, 2013: 455).

Petersen argues that Islam is merely one of the IRW’s characteristics and not embedded in every aspect of its programs, structure, and behaviors (Petersen 2012: 130-131). The IRW’s speech reflects its formula of compromising religious concepts and universal language. It integrates most of the humanitarian universal values into the Islamic value system; as both target human solidarity and dignity. The IRW adopts compulsory Islamic rules, such as free-interest loans and the prohibition of abortion. Otherwise, it evaluates its approach, goals, and programs considering Islamic concepts of *Maslaha* (interest) and *Mafsadah* (corruption) to shape its religiously inspired path. Contrarily to most Islamic FBOs’ leaders, Hany El-Banna, the IRW’s founder, recognized international restrictions imposed after 9/11 as an opportunity that encouraged Islamic FBOs to enhance their professionalism and transparency (Mansour and Ezzat 2009: 134). He strictly distanced the IRW apart from other international Islamic FBOs because some of these organizations were accused of funding terrorist groups. El-Banna benefited from the IRW’s cooperative relations with the British government to promote the organization’s international humanitarian role. Henceforward, it has intensified its *universal* language more than borrowing Islamic terms. Petersen observed a surprising expansion of the IRW’s programs after 9/1; as it turned to be the largest transnational Muslim organization worldwide. During the 1990s, its budget was about ten million dollars, but a few years later its annual budget reached more than 60 million dollars. Internationally welcomed, it has become a leading partner with many international donor organizations (Petersen 2012: 145).

### Altered paths

FBOs share various moralities, which are classified within three major assessment categories: organizational control, expression of religion, and program implementation. The program implementation is examined by investigating the selection of service provided, the integration of religious elements in service delivery, and the voluntary or mandatory participation in specific religious activities (Bielefeld and Cleveland 2013: 446-447). Relevant to my argument, I analyze the two organizations’ operating programs according to the implementation criteria.

#### Provided services

Provided services could be evaluated based on two dimensions: geographical expansion and offered services. The DAS and the IRW operate most of their programs in Muslim-majority countries and stress that they equally provide services for Muslims as well as non-Muslims in targeted societies. Recently, they expanded their programs to a few non-Muslim majority countries. Since its inception, the DAS has exclusively operated its programs in African countries, and later, it launched some activities in Tunisia and Yemen. Thanks to its wealthy society and welfare state, it does not run any domestic programs. Differently, the IRW seeks to expand its programs worldwide, with limited actual expansion beyond the Muslim-majority countries. It also runs a few domestic programs to provide some developmental services. Geographical expansion in both cases reflects the prioritizing of showing solidarity with Muslims. Logistic factors, such as their friendly accessibility and cultural sensitivity favor their work in Muslim-majority societies. Moreover, the IRW joined numerous international partnerships, which targeted Muslim societies, to benefit from its relative advantages. Except for the DAS’s religious-based programs, both deliver social and developmental services for non-Muslims to express human bonds.

Offered services include humanitarian as well as developmental support, although operated programs vary from an organization to the other. The DAS aims to change the African communities to be “free from illiteracy, poverty, and diseases” (Direct Aid Society, 2019). Thus, it mainly provides education, health care, and developmental services. It operates programs to carry out or support undergraduate and graduate education as well as the adult and vocational education. Its developmental programs fund agricultural projects and social care. Health services are provided by permanent and mobile clinics; performing eye surgeries is common in these clinics. Urgent humanitarian relief also delivers health care in targeted communities. Some offered services primarily serve Muslims, with an unclear status of non-Muslims. In Islamic festivals, the DAS distributes *Qurbani* meats, and Ramadan fasting parcels for Muslims (Direct Aid Society Annual Report 2013, 2019). The DAS runs regular proselytization programs, and funds building mosques and religious educational institutes. In 2013, its operated programs include 155 Qur’an competitions, 451 proselytization trips, 83 religious camps, 105 training for preachers, 104 classes for newly converted Muslims, and 767 radio programs (Direct Aid Society Annual Report 2013, 2019)

More widely, the IRW operates humanitarian relief and development programs. It pays special attention to offering urgent humanitarian relief during political conflicts or epidemic diseases. It delivers food parcels, clothing, shelters, and sponsorships of orphans. Furthermore, it establishes developmental programs to raise awareness of reproductive health, support educational services, and fund microcredit projects, especially in the post-wars or natural disasters societies (Mamoun 2010 10). Comparing to the DAS, the IRW runs only a few religious-focused services; it occasionally distributes *Qurbani* meats and Ramadan food parcels. The organization mostly offers general services which are commonly needed and easily adopted in various hosted societies. The IRW argues that neither proselytization nor building mosques are included in its mission. Meanwhile, it focuses on fighting illiteracy, illness, and poverty as its recognized Islamic mission.

#### Religious elements in delivered service

FBOs usually serve religiously similar communities to benefit from their cultural accessibility. Recipient communities generally expect religious aspects of their provided services. Expectations vary among societies and FBOs; it ranges from teaching religious rituals and theology to offering free-interest loans and preventing abortion surgeries. Another implicit expectation is that Islamic FBOs might favor Muslims in the process of delivering services. The frequent selection of Muslim-majority societies to host FBOs’ humanitarian programs shows considerable religious solidarity. Paradoxically, FBOs are currently challenged by calls to secularize their operated programs and de-religious delivered services, although this practically de-activates their religious essence.

Direct religious elements could be traced in many of the DAS-operated programs. It funds religious educational programs, which are opened to Muslims by birth and newly converted ones. These programs teach Islamic rules and enhance religious values. Another special program is dedicated to allowing poor Muslims to travel to *Haj* (traveling to Mecca is the Islamic faith fifth pillar). Also, the DAS established two universities in Kenya and Zanzibar in 1997 and 1998, respectively, which target to increase the number of educated Muslims in these countries, while they still serve non-Muslims. Both offer bachelor’s degrees in applied sciences, humanities, Arabic language, and Islamic Studies^9^. The DAS supports proselytization efforts and train local preachers. Although declared as non-discriminatory, its humanitarian and developmental programs refer to Islamic rules, like offering free-interest loans and opposing abortion.

Closely, the IRW mostly operates in Muslim-majority communities and stresses that it equally offers its humanitarian and developmental services to Muslims as well as non-Muslims. These programs avoid offering interest loans or abortion medical care; however, direct religious services could hardly be traced in this regard. The organization emphasizes on delivering capacity-building services rather than establishing mosques or digging wells (Petersen 2012: 148). Benthall noticed that while the Turkish Red Crescent built mosques in Ache after the Tsunami in 2004, the IRW abstained to take a similar action. Interestingly, Turkey defines itself as a secular state, while the IRW is recognized as an Islamic FBO. The organization claims to avoid any infiltration of religion into relief or developmental goals (Benthall 2008). Its religiously relevant programs, such as the distribution of Ramadan food parcels and *Qurbani* meat, are separated from its other humanitarian or developmental activities. Ironically, the IRW logo contains Islamic symbols more than its counterpart belongs to the DAS. The latter organization clearly expresses its religious affiliations and goals, and easily integrates them in its delivered services, while the former points out to its religious commitments in vague terms and hesitantly combines religious activities in its operated programs; not similar cautions are expressed in its religious-based fund-raising process^10^.

Differences between the two organizations reflect various degrees of the socially constructed secularization and sanctification of humanitarian activities in their headquarter-based societies. The Kuwaiti society explicitly encourages integrating religious dimensions in provided services to acknowledge Islamic solidarity. Contrarily, British society favors a clear distinction between religious connotations and delivered social services to avoid potential discrimination among recipients. Sharing a close understanding of humanitarian actions, promising partnerships between the IRW and other international secular organizations have successfully achieved. The DAS mostly joins regional and international partnerships with other FBOs that follow a similar religious understanding of humanitarian relief. Religiously, Islamic jurisprudence welcomes both approaches, as both show humanitarian solidarity with human beings regardless of their religion. Working mechanisms still depend on the organizations’ social contexts to adjust the level of secularization or sanctification of each society. The only red line in this regard is violating any Islamic compulsory rule. Otherwise, numerous humanitarian and developmental activities are recommended. Each organization could design its favored approach to balance its religious norms and societal mechanisms.

#### Obligatory participation in specific religious activities

Neither the DAS nor the IRW has shown any indication that they require service recipients to participate in specific religious activities. Both stress that they deliver their services equally regardless of race, gender, or religion. The DAS religious proselytization programs acknowledge the freedom of conversion and belief and no evidence was traced linking between the conversion of Islam and delivering the organization’s social services. Adopting the DAS to its current name was justified by the actual expansion of delivered services to cover Muslims and non-Muslims. The IRW does not run any proselytization programs.

## Conclusion: Islamic FBOs’ future—flourishing or falling

The flourishing of FBOs’ humanitarian and developmental roles raises questions about their different approaches, goals, and mechanisms. In Western societies, the influx of immigrants and neoliberal policies encourage the reconsideration of these organizations’ influence. They also maintain an appreciated role in the non-Western communities. I compared two Islamic FBOs: the Kuwaiti Direct Aid Society (DAS) and the UK-based Islamic Relief Worldwide (IRW). While the Kuwaiti religious society significantly enhances the religious aspects of the DAS’s humanitarian and developmental approach, the British secular context lessens the IRW’s religious expressions. The challenge was how to explain their relevancy to the Islamic faith, although of their variant approaches. Historically, each Muslim society used to smoothly develop its formula of charitable activities and humanitarian relief. The “socially-constructed model” refers in this case to any version of Islamic interpretation that adopts a suitable approach, set reasonable goals, and creates friendly mechanisms to its society. As long as Islamic FBOs still seek the *Ultimate* goals of Islamic *Shari’ah*, there will be no issues in the variation of their perspectives, goals, and target audiences of their humanitarian and developmental activities.

Due to the expected countless FBOs’ humanitarian formulas, I predict these organizations’ future in light of four factors: theoretical, socioeconomic, political, and administrative. Theoretically, Muslim scholars should contribute to developing creative balanced religiously inspired and socially constructed FBOs models to ensure their fulfillment of religious commitments and gaining social acceptance. This applies to Muslim minorities in Western secular societies and is also highly recommended for Muslim-majority countries. For the socioeconomic factors, FBOs are expected to raise structural issues, such as social justice, fair conflict resolutions, and empowering disadvantaged groups. Their religious background could inspire them to support social change, instead of replacing withdrawal states in a neoliberal system. Politically, Islamic FBOs’ influence might challenge negative stereotypes, debate controversial issues, and contribute to peaceful and fair conflict settlements. Suggested issues for debate include defining terrorism, conceptualizing humanitarian intervention, and guiding the role of religion in the public sphere^11^. Also, the FBOs’ administrative improvement is needed. A mere religious mission cannot compensate for professional programs, accountable staff, and transparent policies. These actions are required for gaining trust and effectively operate in the hosted communities. Material and spiritual impacts should be measured by innovative indicators to evaluate the programs’ outcomes.

Finally, the religious inspiration does not guarantee success for FBOs, instead, they should address people’s actual concerns and work enthusiastically to achieve factual social change. Otherwise, they will lose their unique character and miss their religious and humanitarian missions.

## Data Availability

Not applicable

## Abbreviations

FBOs: Faith-based organizations
IRW: The UK-based Islamic Relief Worldwide
DAS: The Kuwaiti Direct Aid Society
NGOs: Non-governmental organizations (NGOs)

## Footnotes

1 For examples of this trend, see Act Alliance, *The role of faith-based organizations in humanitarian response: a reflection on the unique role of FBOs in humanitarian crises,* 2015*,* http://actalliance.org/wp-content/uploads/2015/12/WHSSubmission_ACTAlliance_Role-of-FBOs-in-Hum-Response_July2015.pdf, p.3

2 Secular humanitarian organizations were established to engage in relief and development work without preference to any spiritual foundations, but rather, are generally, organized around “universal” human rights principals. For more details, see Act Alliance 2015: 5.

3 For a detailed comparison between FBOs and NGOs, see Clarke et al. 2015: pp.40-46.

4 For additional details, see Mansour and Ezzat 2009: pp.119-122.

5 For more information about the difficulties facing state-affiliated FBOs, see Svoboda et al. 2015; similar case could be noticed in Human Rights Commission, see Petersen 2012-2013, pp.34-36.

6 For more details about the differences among various FBOs, see Clarke and Ware 2015: p.40.

7 An example of these organizations is the Swedish Ibn Rushd Study Organization, see Harding 2013: 345.

8 The International Islamic Relief Organizations was unjustifiably banned in Saudi Arabia at December 2015.

9 For more information about the educational role of the Direct Aid Society, see Yahya 2016: pp.122-131.

10 Both organizations accept Islamic obligatory and voluntary charity. Donors can establish an Islamic endowment to sustainably fund any of their programs. IRW puts a calculator of obligatory charity in its official website (Islamic Relief Worldwide,^2019^)

11 For more recommendations, see *Live online consultation: faith and religion in humanitarian,* 2015: 4; Gibbons 2005: 12-13.

## References

[CR1] Abuarqub, Mamoun (2010) Islamic relief: faith and identity in practice. The Newsletter of INTRAC. 25: p.7. Available Dialog: https://www.intrac.org/wpcms/wp-content/uploads/2017/02/ONTRAC-46-Faith-in-Development-Coping-with-Paradox-2010.pdf. Accessed April 26, 2020.

[CR2] Act Alliance (2015) The role of faith-based organizations in humanitarian response: a reflection on the unique role of FBOs in humanitarian crises. Act Alliance Organization. Available Dialog: http://actalliance.org/wp-content/uploads/2015/12/WHSSubmission_ACTAlliance_Role-of-FBOs-in-Hum-Response_July2015.pdf. Accessed April 26, 2020.

[CR3] Ager, Alastair (2014) Faith, and the secular: tension in realising humanitarian principles. Forced Migration Review. 48: 17-18. Available Dialog: https://www.fmreview.org/faith/ager. Accessed April 26, 2020.

[CR4] Anti-Defamation League (2012) The faith-based initiative and “Charitable Choice”: harmful to religious liberty and civil rights. Anti-Defamation League. Available Dialog: https://www.adl.org/sites/default/files/documents/assets/pdf/civil-rights/religiousfreedom/religfreeres/QA-FaithBasInit-docx.pdf. Accessed April 26, 2020.

[CR5] Bailey, Sarah Pulliam & Michelle Boorstein (2018) Amid Storm Daniels News, Trump announces faith-based effort on national day of prayers. The Washington Post. 3^rd^ May. URL: https://www.washingtonpost.com/news/acts-of-faith/wp/2018/05/03/amid-stormy-daniels-news-trump-expected-to-announce-faith-based-office-on-national-day-of-prayer/?noredirect=on&utm_term=.e80dbf33762a. Accessed on April 26, 2020.

[CR6] Barnett M, Stein JG (2012) Introduction. (In) Michael Barnett and Janice Grass Stein (ed.) Scared aid: faith and humanitarianism. Oxford University Press, New York

[CR7] Benthall, Jonathan (2008) Have Islamic aid agencies a privileged relationship in majority Muslim areas? The case of post-tsunami reconstruction in Aceh. The Journal of Humanitarian Assistance. https://sites.tufts.edu/jha/archives/153. Accessed April 26, 2020.

[CR8] Benthall J, Bellion-Jourdan J (2009) The charitable crescent: politics of aid in the Muslim world, 2nd edn. I. B, Tauris, London

[CR9] Bielefeld W, Cleveland WS (2013) Defining faith-based organizations and understanding them through research. Nonprofit and Voluntary Sector Quarterly. 42(3):442–467. 10.1177/0899764013484090

[CR10] Carlson-Thies SW (2009) Faith-based initiative 2.0: the Bush faith-based and community initiative. Harvard Journal of Law & Public Policy. 23(3):931–947

[CR11] Clarke G (2007) Agents of transformation? Donors, faith-based organisations, and international development. Third World Quarterly. 28(1):77–96. 10.1080/01436590601081880

[CR12] Clarke M, Ware V-A (2015) Understanding faith-based organizations: how FBOs are contrasted with NGOs in international development literature. Progress in Development Studies. 15(1):37–48. 10.1177/1464993414546979

[CR13] Clerkin RM, Grønbjerg KA (2007) The capacities and challenges of faith-based human service organizations. Public Administration Review. 67(1):115–126

[CR14] De Cordier B (2008) Islamic faith-based development organizations in former soviet Muslims environments: the mountain societies development support program in the Rasht Valley, Tajikistan. Central Asian Survey. 27(2):169–184. 10.1080/02634930802355113

[CR15] De Cordier B (2009) Faith-based aid, globalisation, and the humanitarian frontline: an analysis of Western-based Muslim aid organizations. Disasters. 33(4):608–628. 10.1111/j.1467-7717.2008.01090.x 19207537 10.1111/j.1467-7717.2008.01090.x

[CR16] Direct Aid Society (2019) Direct aid register. http://direct-aid.org/cms/en/. Accessed April 26, 2020.

[CR17] Ebaugh HR, Pipes PF, Chafetz JS, Daniels M (2003) Where’s the religion? Distinguishing faith-based from secular social service agencies. Journal for the Scientific Study of Religion. 42(3):411–426. 10.1111/1468-5906.00191

[CR18] Featherstone, Andy (2015) Keeping the faith: the role of faith leaders in the Ebola response. Christian Aid, CAFOD, Tearfund, and Islamic Relief, U.K.

[CR19] Ferris E (2005) Faith-based and secular humanitarian organizations. International Review of the Red Cross 87(858):311–325. 10.1017/S1816383100181366

[CR20] Gibbons, Patrick (2005) Saving lives, alleviating suffering, and maintaining human dignity”: are these goals of humanitarian action universally accepted. (In) Documentation of the Results of the 7^th^ Humanitarian Congress Theory and Practices of Humanitarian Action. Is humanitarianism universal? Medical relief between ambition, principals, and reality. Berlin. November 18-19, 2005.

[CR21] Harding T (2013) Faith-based organizations and the secular state: the establishment of a Muslim Study Association in Sweden. Journal of Muslim Minority Affairs 33(3):341–355. 10.1080/13602004.2013.863077

[CR22] Islamic Relief Worldwide (2019). Islamic Relief Worldwide register. http://www.islamic-relief.org/. Accessed April 26, 2020.

[CR23] Issac JC (2003) Faith-based initiatives: a civil society approach. The Good Society. 12(1):3–10. 10.1353/gso.2003.0027

[CR24] James R (2011) Handle with Care: Engaging with faith-based organisations in development. Development in Practice. 21(1):109–117. 10.1080/09614524.2011.530231

[CR25] Kemper RV (2006) Anthropological perspectives on faith-based organizations. Urban Anthropology and Studies of Cultural Systems and World Economic Development. 35(2/3):141–153

[CR26] Kirmani N, Khan AA, Palmer V (2008) Does faith matter? An examination of Islamic relief’s work with refugees and internally displaced persons. Islamic Relief Worldwide, Birmingham, UK. 10.1093/rsq/hdn032

[CR27] Live online consultation: faith and religion in humanitarian (2015) Summary Report of the Online Consultation Event in Support of the World Humanitarian Summit. International Association of Professionals in Humanitarian Assistance and Protection (PHAP). Brussel, Belgium.

[CR28] Mansour, Khaled, and Heba Raouf Ezzat (2009) Faith-based action in development and humanitarian work. (In) Ashwani Kumar, Jan Art Scholte, Mary Kaldor, Marlies Glasius, Hakan Seckinelgin, Helmut Anheier (eds.). Global Civil Society**.** London: Sage. pp.118-145. DOI: 10.4135/9781446269275.n7

[CR29] Petersen MJ (2012) Islamizing aid: transnational Muslim NGOs After 9.11. Voluntas. 23:126–155. 10.1007/s11266-011-9185-5

[CR30] Petersen MJ (2012-2013) Islamic or Universal human rights? The OIC’s independent permanent human rights commission. Danish Institute for International Studies Report 2012-03. Danish Institute for International Studies, Copenhagen: Denmark

[CR31] Svoboda E, Zyck SA, Osman D, Hashi A (2015) Islamic humanitarianism? The evolving role of the organization for Islamic cooperation in Somalia and beyond. Humanitarian Policy Group Working Papers. Humanitarian Policy Group, London: UK

[CR32] Yahya YAM (2016) Dor Jamiyyat Al-Awin Al-Mubashir fi Al-Ta’lim Al-Ali: Halati Kenya wa Zanjabar (Role of the Direct Aid Society in higher education: the cases of Kenya and Zanzibar). Qira’at Africiyya (African Readings) XI(27):122–131

[CR33] Zehavi A (2008) The faith-based initiative in comparative perspective: making use of religious providers in Britain and the United States. Comparative Politics. 40(3):331–351

